# Probabilistic population aging

**DOI:** 10.1371/journal.pone.0179171

**Published:** 2017-06-21

**Authors:** Warren C. Sanderson, Sergei Scherbov, Patrick Gerland

**Affiliations:** 1 Department of Economics, Stony Brook University, Stony Brook, NY, United States of America; 2 International Institute for Applied Systems Analysis, World Population Program, Wittgenstein Centre for Demography and Global Human Capital (IIASA, VID/ÖAW, WU), Schlossplatz 1, Laxenburg, Austria; 3 Austrian Academy of Science, Vienna Institute of Demography, Vienna, Austria; 4 Russian Presidential Academy of National Economy and Public Administration (RANEPA), Moscow, Russian Federation; 5 United Nations Organization, Department of Economic and Social Affairs (DESA), Population Division, Two UN Plaza, Room DC2-1934, New York, NY, United States of America; University of West London, UNITED KINGDOM

## Abstract

We merge two methodologies, prospective measures of population aging and probabilistic population forecasts. We compare the speed of change and variability in forecasts of the old age dependency ratio and the prospective old age dependency ratio as well as the same comparison for the median age and the prospective median age. While conventional measures of population aging are computed on the basis of the number of years people have already lived, prospective measures are computed also taking account of the expected number of years they have left to live. Those remaining life expectancies change over time and differ from place to place. We compare the probabilistic distributions of the conventional and prospective measures using examples from China, Germany, Iran, and the United States. The changes over time and the variability of the prospective indicators are smaller than those that are observed in the conventional ones. A wide variety of new results emerge from the combination of methodologies. For example, for Germany, Iran, and the United States the likelihood that the prospective median age of the population in 2098 will be lower than it is today is close to 100 percent.

## Introduction

Population aging poses widely discussed policy challenges [1–4]. The United Nations publishes probabilistic forecasts of three measures of population aging, the old-age dependency ratio, the total dependency ratio, and the potential support ratio [5]. These provide information about the likely extent and uncertainty of population aging on the national level and so provide the basis for policy and academic analyses.

Probabilistic population forecasts were motivated by Keyfitz [6]. Keyfitz wrote:

“Demographers can no more be held responsible for the inaccuracy in forecasting population 20 years ahead than geologists, meteorologists, or economists when they fail to announce earthquakes, cold winters, or depressions 20 years ahead. What we can be held responsible for is warning one another and our public what the error of our estimates is likely to be.” (p. 579).

There is now an extensive literature of probabilistic forecasting [7–18]. All the probabilistic measures of aging produced by the United Nations assume that the threshold of old age is a fixed chronological age, regardless of time, place, education, or other characteristics of people. Ryder [19](page 16) questioned this assumption. He wrote:

“To the extent that our concern with age is what it signifies about the degree of deterioration and dependence, it would seem sensible to consider the measurement of age not in terms of years elapsed since birth but rather in terms of the number of years remaining until death.”

Ryder suggested the old age threshold be defined on the basis of some plausible remaining life expectancy rather than any specific chronological age. Sanderson and Scherbov [20,21] extended and generalized Ryder’s idea. They defined characteristic-equivalent ages as chronological ages at which a measure of some characteristic is held constant. We call the ages, that are obtained when life expectancy is the characteristic that is held constant, prospective ages and measures that use them prospective measures of population aging. In this paper, we merge two methodologies, probabilistic population forecasting based on a Bayesian hierarchical model [9] and prospective ages to produce new probabilistic forecasts of aging.

New measures of population aging are useful because tomorrow’s older people will not be like today’s. They may well have longer life expectancies [22,23], better cognition [24], better education [25], and fewer severe disabilities [26]. In most OECD countries, the labor force participation of people 65+ years old is increasing [27] as are the ages at which people can receive a normal national pension [28,29]. Since changes in the characteristics of people are ignored in the conventional measures of aging, they become more outdated with the passage of time. The use of prospective ages is one way to create measures of aging that are more in line with observable changes.

Prospective ages can be used in a wide variety of contexts in the study of population aging. Here we use them in two ways. First, we follow Ryder and define the threshold of old age based on a remaining life expectancy rather than a fixed chronological age. We chose a remaining life expectancy of 15 years. That was the life expectancy at age 65 in many low mortality countries around 1970.

**Fig 1:**
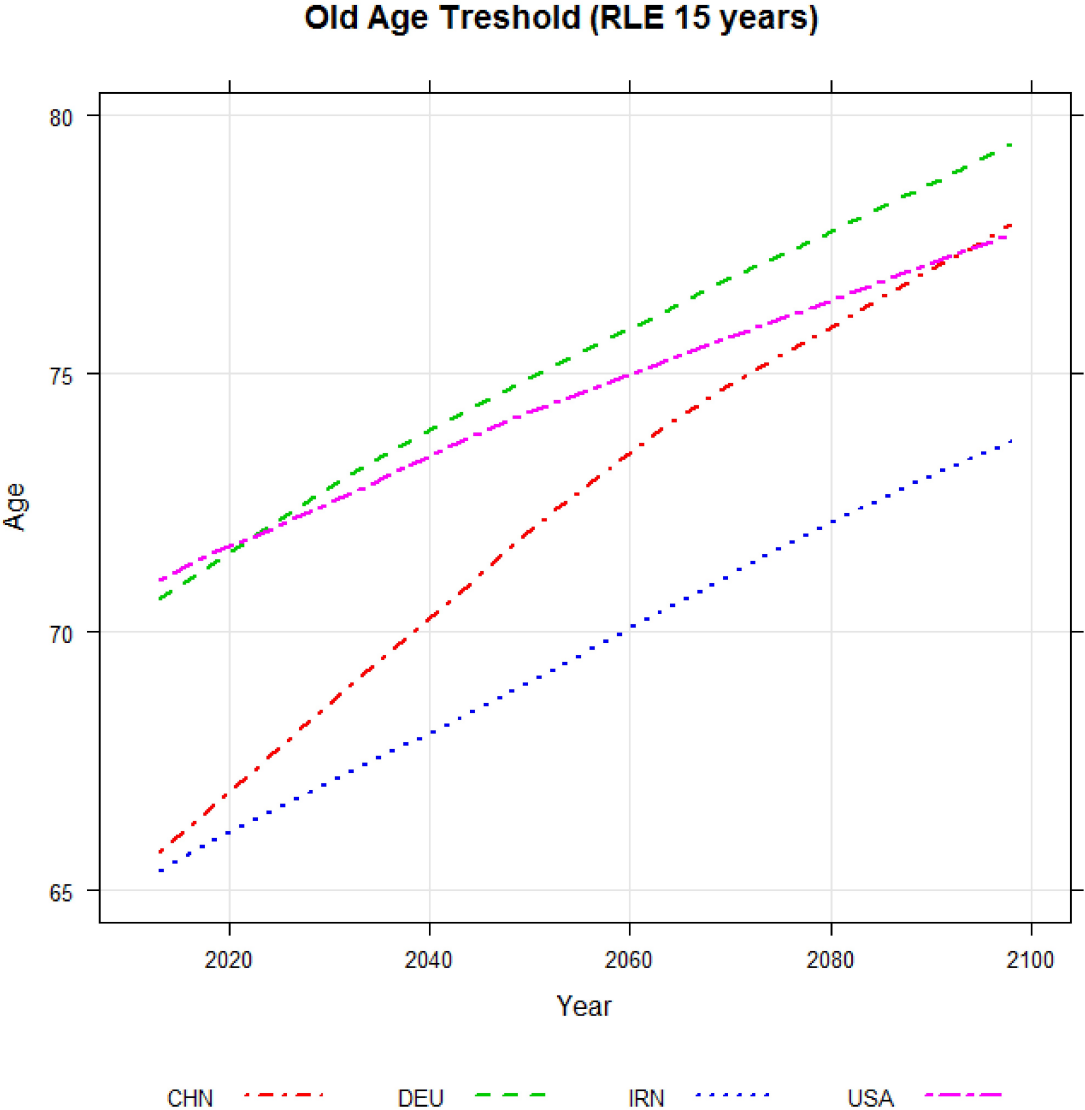
Old age threshold based on a remaining life expectancy of 15 years. Source: UN (2015) and authors’ calcuations.

In Fig 1, we show estimated and forecasted old age thresholds based on a remaining life expectancy of 15 years for China, Germany, Iran and the US for the years 2013 through 2098. In 2013, the old age threshold was 66 in China and 72 in Germany. By 2098, the old age threshold is forecasted to increase to 79 in Germany and 77 in China.

In the second application of prospective ages we modify the conventional median age [20,21]. Instead of reporting median ages in terms of chronological ages, we report them in terms of prospective ages. For example, Table 1 shows the median age and the prospective median age of the US population from 2013 to 2098, as well as the remaining life expectancy at the median age. In Table 1, we use the life table of the US in 2013 as a reference. The prospective median age standardizes the median age for changes in life expectancy. In particular, the prospective median age is the age in the reference life table where remaining life expectancy is the same as at the median age in the specified year. The median age in the US in 2013 was 37.6 years. At that time the remaining life expectancy of people 37.6 years old was 43.0 years. In 2098, the median age is forecasted to increase by 6.9 years to 44.5 and the remaining life expectancy is also expected to rise from 43.0 to 45.4. In 2013, the base year for the calculation of the prospective median age, people had a remaining life expectancy of 45.4 years at age 35.1. Therefore, the prospective median age in the US in 2098 is 35.1. People in the US at the median age in 2098 are expected to be older than the people at the median age in 2013 but, nevertheless, have a longer remaining life expectancy.

**Table 1:** Median age, prospective median age, and remaining life expectancy at the median age: USA, 2013–2098.

country	year	Median Age (MA)	Prospective Median Age (PMA)	Remaining Life Expectancy at the Median Age (RLE at MA)
USA	2013	37.6	37.6	43.0
USA	2018	38.3	37.7	42.9
USA	2023	38.9	37.7	42.9
USA	2028	39.7	37.8	42.8
USA	2033	40.4	37.9	42.7
USA	2038	41.0	37.8	42.8
USA	2043	41.3	37.5	43.1
USA	2048	41.6	37.1	43.5
USA	2053	41.8	36.7	43.8
USA	2058	41.9	36.4	44.2
USA	2063	42.2	36.1	44.4
USA	2068	42.6	36.0	44.5
USA	2073	43.0	35.9	44.6
USA	2078	43.3	35.7	44.7
USA	2083	43.6	35.5	44.9
USA	2088	43.9	35.4	45.1
USA	2093	44.2	35.2	45.3
USA	2098	44.5	35.1	45.4

Source: UN (2015) and authors’ calculations

## Materials and methods

We drew for each country a systematic subsample of one thousand random trajectories from the 10,000 that were the basis of the UN’s 2015 probabilistic population projections. Each trajectory included the age structure of the population at 5 year intervals starting in 2015 and abridged life tables for five year intervals from 2015–2020 to 2095–2100 combining data for both sexes. Conventional measures were computed at the midpoint of the 5 year intervals and prospective measures were computed applying the corresponding life tables to those populations.

The old age threshold used in the computation of the prospective proportion of the population who are old and the prospective old age dependency ratio is derived from the equation:

$$oat_{j,t}=e_{j,t}^{-1}\left(15\right),$$

where *oat*_*j*,*t*_ is the old age threshold in country *j* in year *t* and $e_{j,t}^{-1}\left(15\right)$ is the age in the life table for country *j* in year *t* where remaining life expectancy is equal to 15 years.

The prospective median age in country *j* in year *t* is derived from the equation:

$$pma_{j,t}=e_{j,2013}^{-1}[e_{j,t}\left(ma_{j,t}\right)],$$

where *pma*_*j*,*t*_ is the prospective median age in country *j* in year *t*, *ma*_*j*,*t*_ is the median age in country *j* in year *t*, *e*_*j*,*t*_(*ma*_*j*,*t*_) is the life expectancy in the life table for country *j* and year *t* at the median age of the population in that year, and $e_{j,2013}^{-1}\left[e_{j,t}\left(ma_{j,t}\right)\right]$ is the age in the country’s life table of 2013, where remaining life expectancy is the same as at the median age in year *t*.

## Results

**Fig 2:**
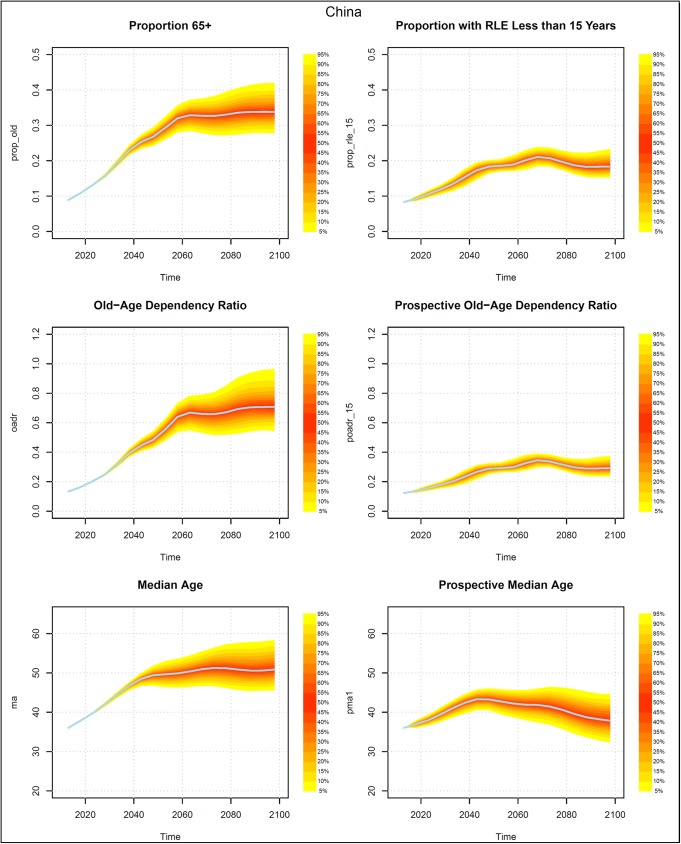
Probabilistic forecasts for three aging measures based on chronological ages and three based on prospective ages, China 2013–2098. Source: UN (2015) and authors’ calculations.

**Fig 3:**
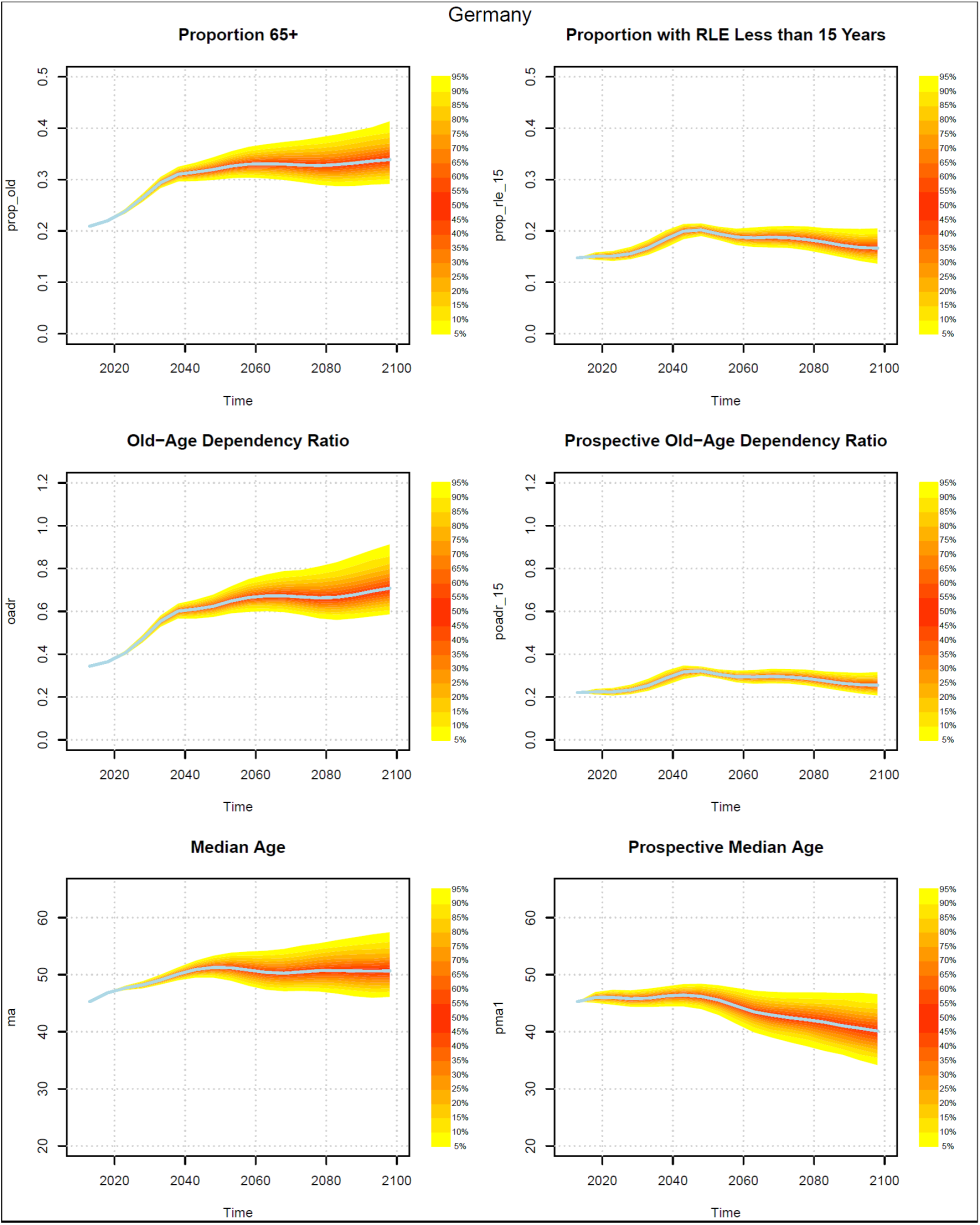
Probabilistic forecasts for three aging measures based on chronological ages and three based on prospective ages, Germany 2013–2098. Source: UN (2015) and authors’ calculations.

**Fig 4:**
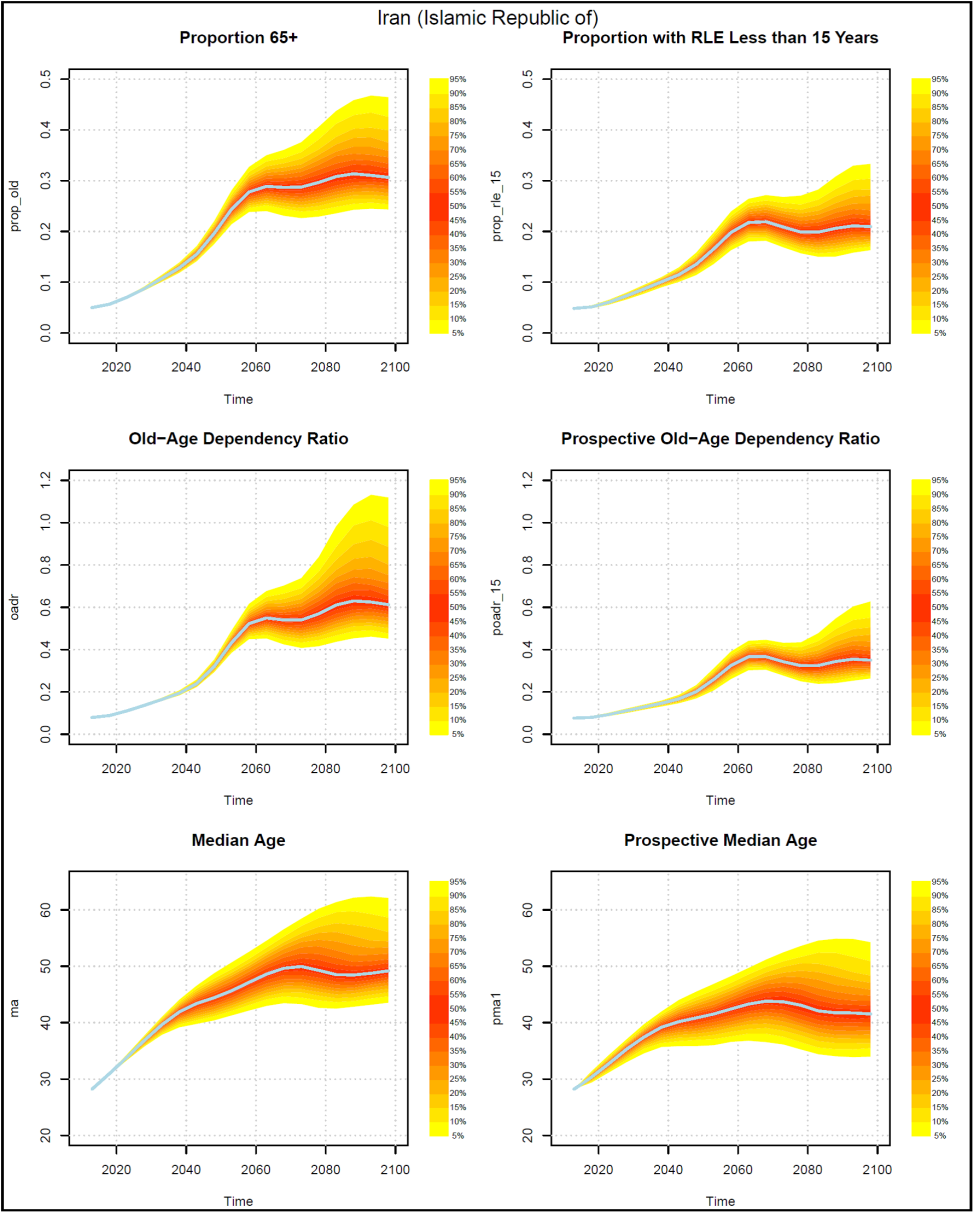
Probabilistic forecasts for three aging measures based on chronological ages and three based on prospective ages, Iran 2013–2098. Source: UN (2015) and authors’ calculations.

**Fig 5:**
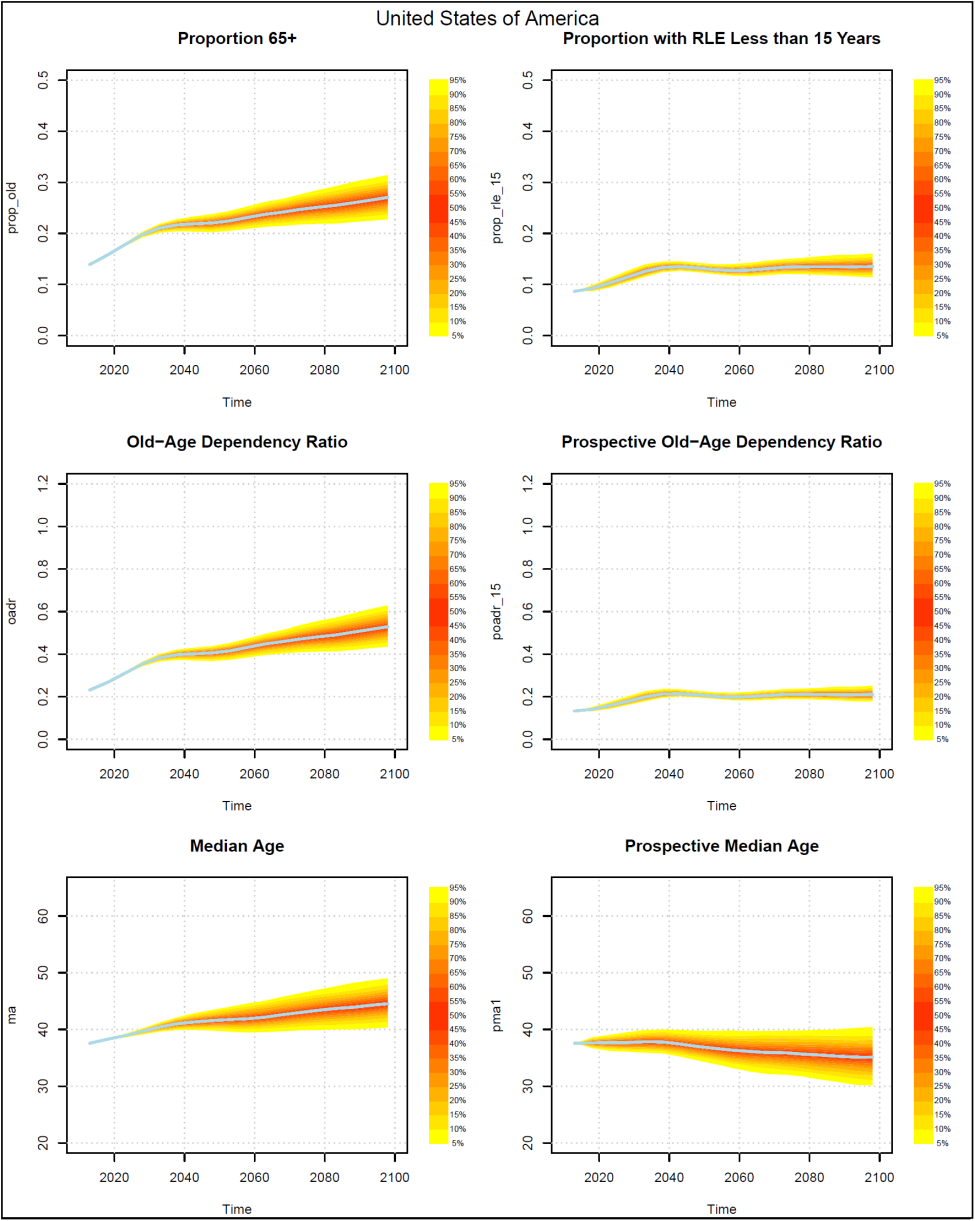
Probabilistic forecasts for three aging measures based on chronological ages and three based on prospective ages, USA 2013–2098. Source: UN (2015) and authors’ calculations.

Figs 2–5 present measures of population aging for four countries, China, Germany, Iran, and the US. Each figure has six panels. The panels on the left-hand side show three measures of aging that are based on chronological ages. The topmost shows the probabilistic distribution of the proportion of the population who are 65+ years old. The middle graph presents the distribution of the old age dependency ratio (OADR), defined as the ratio of people 65+ years old to those 20–64 years old. The bottom graph shows the distribution of the median age of the population.

The graphs on the right side present analogous measures that utilize prospective ages. In the topmost graph the onset of old age is defined using a prospective age instead of the fixed age 65. In that graph, the threshold of old age is set to the age at which remaining life expectancy was 15 years. In the middle graph on the right, the prospective old age dependency ratio is defined as the ratio of people at or above the prospective threshold age to the number of people between 20 and the prospective threshold age. The prospective median age in the bottom right graph is the age in 2013 (taken here as the base year) where remaining life expectancy is the same as at the median age in the indicated year.

Prospective proportions of the population who are old and prospective old age dependency ratios (POADR) are lower in 2013 than their counterparts that use age 65 as the old age threshold because the age at which remaining life expectancy was 15 years was above age 65. The prospective measures also grow less rapidly. The probabilistic forecasts also reveal a difference in the standard deviations of the forecasts.

The standard deviations of the forecasts of the prospective proportion of the population who are old and the prospective old age dependency ratios (POADR) are less than their counterparts that do not use prospective ages. In Germany and the US, the standard deviation of the POADR in 2058 was 0.01, while the standard deviations of their OADRs in that year were 0.05 and 0.03 respectively. In China, the analogous numbers are 0.03 for their POADR and 0.06 for their OADR. In the case of Iran the difference between the standard deviation of the POADR and the OADR is smaller, being 0.04 and 0.05 respectively.

The standard deviations of the POADR remain relatively small even to the end of the century. In 2098, the standard deviation was 0.05 for China, 0.03 for Germany, and 0.02 for the US. Indeed, in the US, the 90 percent prediction interval for the POADR in 2098 was between 0.18 and 0.25. In Iran, uncertainty with respect to the POADR in 2098 was much higher than in the other three countries. The standard deviation there was much higher, 0.13.

The distributions of the four measures look different for Iran than for the other three countries. Iran experienced one of the most rapid decreases in fertility in history during the 1980s [30]. The resulting waves in the population age structure propagate over time and produce the uncertainties that we see in Fig 4, which are larger than for any of the other countries. China also had a rapid fertility decline, which occurred somewhat earlier than Iran’s, which lead to an irregular age distribution, as well. The large irregularities in the age structures in Iran and China contributed to the large observed uncertainties in the prospective measures.

Turning to the median age and the prospective median age, we see that while the median age either rises or remains roughly constant over time, the prospective median age actually falls. The decline begins earliest in the US, where the peak prospective median age is 37.8 at around 2038. The prospective median age then falls to 35.0 by 2098. The decline in prospective median age begins slightly later in Germany where it falls from 46.5 in 2043 to 40.1 in 2098. The decline in China begins around 2048 and around 2073 in Iran. By 2098, Iran has the highest median age and prospective median age among the four countries. In contrast to the case for the OADR and the POADR, the standard deviations of the prospective median age tend to be about the same as for the median age.

## Discussion

Each of the trajectories that we use is computed using assumptions about the paths of fertility and mortality rates. In the four countries that we are considering infant and child mortality rates are already quite low at our starting date in 2013, so most of the increase in life expectancy arises because of future increases in survival at upper ages. Some probabilistic trajectories will tend to have higher life expectancies and other lower ones. The life expectancies are auto-correlated, so that a trajectory with a higher than average life expectancy in one year has a greater probability of having a higher than average life expectancy in the following year. On trajectories that, on average, have higher life expectancies, the number of people aged 65+ is higher and therefore, so is the proportion of the population 65+ and the old age dependency ratio.

When we consider the prospective proportion of the population who are old and the prospective old age dependency ratio, there is another factor at work. Populations with higher numbers of 65+ year olds have, on average, higher life expectancies, and, therefore, higher old age thresholds. Higher old age thresholds decrease both the prospective proportion old and the old age dependency ratio. Thus, higher life expectancies produce two offsetting effects on the prospective measures.

The same factors that affect the relationship between the OADR and the POADR affect the relationship between the median age and the prospective median age. One difference is that the prospective median age uses the median age as an input, while the POADR does not use the OADR as an input. Since the prospective median age uses the median age as an input, the distribution of median ages in a given year is one component of the distribution of prospective median ages. In addition to the uncertainty in the median age, the prospective median age also is affected by variations in prospective ages resulting from differences in life tables.

We can draw several conclusions from this analysis. First, prospective measures of aging are both lower than their conventional counterparts and they increase more slowly or even decrease. Second, prospective measures generally have smaller standard deviations than those based only on chronological age.

The third conclusion is that most of the aging that we measure in China, Germany, and the US occurs between now and around 2040. In the case of Iran, it continues to around 2060. In Germany, the prospective old age dependency ratio is forecasted to be around 0.22 in 2018. By 2038, the median prospective old age dependency ratio rises to 0.29 with a 90 percent prediction interval from 0.26 to 0.32. By 2098, the median prospective old age dependency ratio falls to 0.26 with a 90 percent prediction interval from 0.22 to 0.32. Put differently, in 2018, we would expect there to be around 4.5 people from age 20 to the old age threshold per person at or above the old age threshold. By 2038, when aging measures would nearly be at their maximum, the 90 percent prediction interval is between 3.1 and 3.8.

The probabilistic forecasts show that it is highly likely that the prospective median age of the population of Germany and the US will be lower in 2098 than it is today. Even in China there is around a 50 percent chance that the prospective median age will be lower at the end of the century than it is today. It is possible that people’s concern about the future is related to the number of additional years they expect to live. Lower prospective median ages in 2098 than now indicate that the people at the median age will have even more years of additional life ahead of them than people at the median age have currently.

Policy-makers and others who want to understand and prepare for the future need appropriate data to guide them. Measures of population aging that do not take the changing characteristics of people into account do not provide this. Probabilistic prospective measures of population can provide the kinds of information that is needed.
